# In Nature's School

**Published:** 1889-08-31

**Authors:** 


### The Doctor's Pulpit.

### IN NATURE'S CHOOL.

### "Dodging" the Weather.

Simple-minded people, who are always the best, do not like the idea of "dodging" anything. The word itself is objectionable as well as the idea; but an attempt to find another which expresses what the writer wants to express so exactly and precisely as "dodging" having completely failed, there is no choice but to let the word stand and to make the best of it. In bad seasons farmers know very well the meaning of "dodging the weather." Some of their crops are exceedingly dependent on weather, and may be completely ruined by too prolonged exposure to rain. Everybody knows that this is so in the case of hay. Hops also, as hop growers know to their cost, are very "weathery" subjects, and so, in a less degree, are all kinds of corn. The difficulty in getting hay and corn reaped in showery weather consists in this, that a certain degree of dryness must be attained before either the one or the other can be stacked with safety. To pile up wet hay into a huge stack not only means the risk of a fire from spontaneous combustion, but also the absolute certainty of much of it becoming so mouldy and malodorous that the cattle will not eat it, and if they did it would be almost as bad as poison to them. The farmer has therefore to watch the clouds early and late for weeks together, to consult his weather glass a dozen times a day, to examine his crop, whether it be ripe for cutting, or dry enough for carrying, and to get it reaped in any way he can, sometimes a few loads at a time, until the whole is cleared. Even in a rainy season many a man will "dodge the weather" so successfully that he will have his stack-yard filled and his stubbles cleared in a very few days over the usual period of hay and corn harvest. Others, again, are so wanting in skill and watchfulness, so slow at seizing opportunities, that they have their hay-fields unreaped in September, and the corn still standing out in the dense November fogs. No man needs to be told that bankruptcy and ruin lie that way.

What the farmer has to do in the department of agriculture the town man has to do in the department of health. To the inhabitant of the large city the weather is but one enemy among many he has to contend with, and to evade or resist. In the case, however, of rains, snows, and intense heats evasion is better than resistance. Very few men bred in towns can stand a "drenching" without running serious risks. The countryman may get wet, and if he be young and strong may let his clothes dry on him with little danger. But that is because, not only is he initially stronger, but he is moving about all day in the open air, and so keeps up, by exercise, the animal heat of his body. The town man, on the other hand, when he gets wet goes and seats himself in an office. The temperature of the body is soon lowered, for the double reason that he is not keeping himself warm by movement, and that the cold rain in his soaked clothing rapidly chills the surface. One man in twenty may be strong enough to stand even this unharmed; but the other nineteen will be in imminent danger of sore throat, inflammation of the lungs, rheumatic fever, or some other similar painful and dangerous disease.

Young men are ashamed to be seen taking care of themselves. They hate what they call "molly-coddling." No doubt it is much more gratifying to a man's pride to be able to despise small cares than to be worried by them. But the really sensible and practical young man will open his eyes to facts. He is a City man, perhaps not by choice, but by necessity. But, whatever the reason, the fact remains that he is a City man. And being a City man, shut up in an office all day, never exposed to the hardening atmosphere, and not building up his constitution by strenuous and continued physical labour, he becomes, whether he likes it or not, much less physically robust, and much less able to withstand the changes of temperature and moisture in the air, than he would have been had he been still a countryman. The points for him to consider are, whether he will learn something from the experience of others or be guided solely by his own. If he consents to take warning and guidance from others, not only may he save himself many pains, much disablement and much expense, but he may find himself when he is no longer young full of robust health, and physically able to put to the most profitable and effective use the large stores of knowledge and experience which he has been gathering for years. If, on the other hand, he despises what he considers the "croakings" of persons older or better instructed than himself, he will undoubtedly learn in time and probably at serious cost. His experience may be bought at the expense of a typhoid fever, a chronic bronchitis, a worrying lung affection, or a disabling and life-shortening injury to the heart.

Life in large towns requires much more care and skill than life in the country. The dangers are far more numerous; the power of resistance in men and women is far less. Because a particular individual is born healthy, and remains strong until manhood, having by good fortune escaped rheumatism, lung affections, diphtheria, typhoid fever, and the thousand and one enemies that lie in wait in almost every town house and street, therefore he looks upon all these evils as mere names, and not as realities to be watched for and guarded against as deadly foes. But when a man has experienced in his own person the attack of any one of these scourges, he knows what it is to lie helpless and passive, and to wait for recovery or death. If, moreover, fever, consumption, or other fatal disease, have once and again carried off before his eyes one or more members of his own household, he knows by the painful sense of grief and loss what potential dangers lie around him on all hands. Many a man cannot respect what he does not fear, and many need the spur of fear before they will learn some of the most important lessons that life has to offer. The farmer "dodges" the weather in order that he may save his yearly crops. The town man must learn, for his life's sake, to avoid all kinds of danger, not only from weather, but also from bad smells, impure water, ill-chosen diet, unhealthy habits, overwork, late hours, and a thousand other things. Life in town must be regarded as a game of skill rather than as a game of chance. He who can preserve sound mental and bodily health for the longest period is most likely to win the highest prizes. For the prizes of life that are really worth having are seldom obtained by a mere stroke of luck. Usually they have to be toiled for strenuously, and waited for patiently. He whose power both of toiling and waiting are exhausted by illness or bad habits may find himself flagging at the very moment when a spurt is needed, or dropping down at the roadside just when the goal is in sight.

There is nothing that appeals to the heroic in learning how to avoid the dangers of town life and how to take advantage of its opportunities of health. No poet sings of the risks of a shower, no orator dilates on the death-dealing potency of a bad smell. Most true! Still, inasmuch as the poet sings but poorly when his health is broken down, and cannot sing at all when he is dead, even he may learn the art of preserving his own bodily energy as the necessary condition of the continuance of his work. It is not in itself a selfish thing to have an intelligent care for health, though many valetudinarians are intolerable nuisances. But if a man cares for his business, which is but the means of life, surely he may care as much and even more for his physical strength, which is a part of life. A great many persons have much to learn, even of the very elements of value. A rearrangement of popular ideas is urgently needed before people can be said to be properly adjusted to their modern environments. Health must be placed on a higher level, and the means that promote it must be much more widely and diligently studied. As character is the basis of all that is successful and worthy in the moral sphere, so are physical robustness and staying power the indispensable conditions of success and happiness in the business of life. Since health is the fundamental necessity of every kind of well-being and success, the thorough study of it should be one of those practical duties which no man feels himself at liberty to leave undone.

